# Stroke Prevention With Oral Anticoagulants in High-Risk Atrial Fibrillation in an Aging Population

**DOI:** 10.1016/j.jacadv.2025.101757

**Published:** 2025-05-09

**Authors:** Ken Okumura, Masaharu Akao, Shinya Suzuki, Takeshi Yamashita

**Affiliations:** a Division of Cardiology, Saiseikai Kumamoto Hospital Cardiovascular Center, Kumamoto, Japan; b Department of Cardiology, National Hospital Organization Kyoto Medical Center, Kyoto, Japan; c The Cardiovascular Institute, Tokyo, Japan

**Keywords:** atrial fibrillation, bleeding, elderly, frailty, oral anticoagulation, renal impairment

## Abstract

Aging societies will pose unique health challenges in the near future. Elderly and very elderly patients often have complex medical needs, including comorbidities and polypharmacy. Contributing to this, atrial fibrillation (AF) is common among elderly patients. Direct oral anticoagulants are widely used to prevent stroke in patients with AF. However, patients in randomized controlled trials tend to be younger than many patients with AF and may not have the complicating factors that can influence treatment decisions. In this review, we summarize what is currently known about direct oral anticoagulants in elderly (age 65-79 years) and very elderly (age ≥80 years) patients with AF, and highlight the remaining gaps in the literature. Although further randomized controlled trials are needed, the ELDERCARE-AF (Edoxaban Low-Dose for Elder Care Atrial Fibrillation Patients) trial may have contributed to filling these gaps.

## Highlights

• Many societies are aging, and very elderly patients have complex medical needs.
• Outcomes of each direct oral anticoagulant treatment in very elderly patients are still unclear.
• Subanalyses of trials and ELDERCARE-AF may have provided some insights.

Atrial fibrillation (AF) is the most common cardiac arrhythmia in the elderly. As the global overall population is aging, the incidence and prevalence of AF have risen markedly in recent decades.^1^^,^^2^ In Europe, 18 million people are expected to have AF by 2060, primarily considered to be the result of an increase in patients with AF aged ≥80 years.^3^^,^^4^ Elderly (age 65-79 years) and very elderly people (age ≥80 years) commonly have multimorbidities, which can lead to challenges in managing treatment strategies.^5^ AF management is particularly important because of its risk for stroke and systemic thromboembolism.^6^ A widely used therapy to prevent stroke and systemic thromboembolism in patients with AF without rheumatic mitral stenosis or mechanical heart valves (hereafter referred to as AF) is direct oral anticoagulants (DOACs).^7^ With several advantages over warfarin, including fewer food–drug interactions, patients receiving DOACs have a significantly lower incidence of all-cause mortality and major bleeding than patients receiving warfarin.^8^, ^9^, ^10^, ^11^, ^12^ However, patients in many trials comparing DOACs with warfarin are often younger than patients with AF in actual clinical practice, and there remains a paucity of evidence in very elderly patients.^13^ Subgroup analyses of clinical trials have attempted to address these concerns, yet data in very elderly patients and those with a history of bleeding, renal impairment, cognitive impairments, or frailty remain insufficient.^13^

Understanding the risks and benefits of treatments is increasingly important in the context of aging societies (Central Illustration). This state-of-the-art review will examine the current evidence regarding the use of DOACs to improve health outcomes in elderly and very elderly patients with AF. The search terms used were “anticoagulation,” “atrial fibrillation,” “elder/aging,” or “outcome” in Medline, PubMed, EMBASE, and Scopus.

**Central Illustration:**
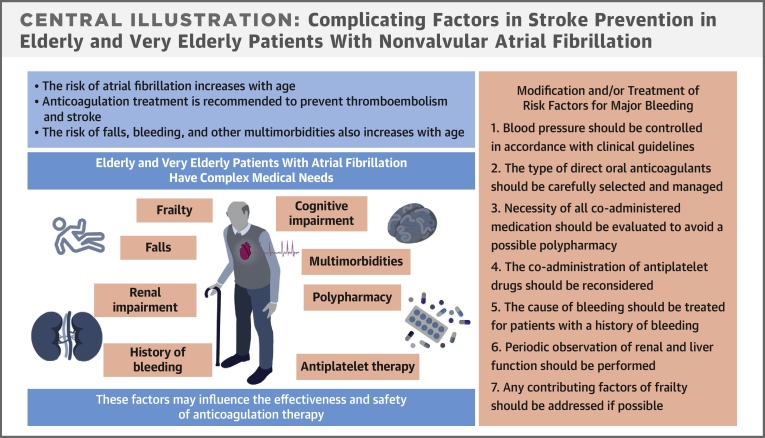
Complicating Factors in Stroke Prevention in Elderly and Very Elderly Patients With Nonvalvular Atrial Fibrillation

## Pharmacokinetic changes in elderly patients

With aging, changes in pharmacokinetics and pharmacodynamics occur because of alterations in end-organ function, receptor sensitivity, homeostasis patterns, concurrent medication use, and the complexity of concomitant diseases.^14^ Renal and liver functions play a crucial role in DOAC metabolism and excretion. Changes in body size are also related to the volume of distribution, thereby affecting the blood concentration of DOACs.^15^ Clinicians should carefully consider the potential increase in drug plasma concentrations and associated risk of adverse events when treating elderly patients.

### Renal function

Renal function progressively declines as age increases because of diminishing renal mass and glomerular filtration rate. Beyond the age of approximately 40 years, this decreases at a rate of around 1 mL/min/1.73 m^2^ per year.^16^ This reduction prolongs the plasma half-life of renally excreted drugs, leading to an increased steady-state concentration.^17^ Several models have demonstrated decreased renal clearance of DOACs in older patients with severe renal impairment (creatinine clearance [CrCl] <30 mL/min) compared with younger healthy participants (32%-66% for edoxaban, 45%-63% for apixaban, 25%-52% for dabigatran, and 17%-40% for rivaroxaban).^18^

### Hepatic function

Hepatic blood flow decreases with age by approximately 40%, reducing drug metabolism.^19^ Liver mass also decreases, reducing the activity of hepatic microsomal enzymes and prolonging the half-life of hepatically metabolized drugs. This reduced hepatic function is exacerbated by polypharmacy, which can saturate the already diminished enzyme capacity.^14^ In a population pharmacokinetic study, hepatic clearance of rivaroxaban decreased by 10% in patients with elevated total bilirubin (20 μmol/L) compared with patients with normal liver function;^20^ however, this was not supported by other studies.^18^

### Body size and composition

Age-related changes in body size and composition, such as a reduced bodyweight and muscle mass loss, may influence DOAC concentration. Bodyweight is closely related to the volume of distribution and serves as one of the dose-reduction criteria for edoxaban and apixaban. Patients with a low bodyweight (≤50 kg) have 27% higher mean peak serum concentrations of apixaban and 20% greater area under the curve values than patients weighing 65 to 85 kg.^21^ A study reported that older age in patients with AF and reduced lean muscle mass were associated with higher DOAC levels at trough (OR per 1 kg reduction, 1.23).^22^

## Complicating factors in treating elderly and very elderly patients with AF

Increased age, frailty, renal impairment, bleeding history, cognitive decline, and the presence of multimorbidities and polypharmacy—including concomitant antiplatelet therapy—are considered complicating factors in elderly and very elderly patients with AF.^13^

### Increased age

Elderly patients with AF have both a higher risk of thromboembolism^23^ and a higher risk of bleeding.^24^ All pivotal DOAC trials demonstrate consistent efficacy of each DOAC vs warfarin in stroke prevention across different age strata, whereas the safety profile of each DOAC vs warfarin is not necessarily consistent across age groups (Table 1).^24^, ^25^, ^26^, ^27^ In patients aged ≥75 years, compared with warfarin, dabigatran 150 mg twice a day showed a trend toward higher risk of major bleeding, and dabigatran 110 mg twice a day showed a similar bleeding risk.^25^ Rivaroxaban vs warfarin showed a similar risk of major bleeding in patients aged ≥75 years.^26^ In contrast, apixaban vs warfarin showed a significantly lower rate of major bleeding in patients aged ≥75 years.^27^ Both higher (standard) and lower (half) doses of edoxaban vs warfarin showed a significantly lower risk of major bleeding in those aged ≥75 years.^24^

**Table 1:** Bleeding Risks of Each DOAC in Patients Aged ≥75 Years

	Dose	Major Bleeding		Intracranial Bleeding		Fatal Bleeding		First Author/Study Design	Study Period
		%/Year	HR^d^ (95% CI)	%/Year	HR^d^ (95% CI)	%/Year	HR^d^ (95% CI)		
N = 7,238 Dabigatran 150 mg bid, n = 2,466 Dabigatran 110 mg bid, n = 2,349 Warfarin, n = 2,423	Dabigatran 150 mg bid Dabigatran 110 mg bid Warfarin	5.10 4.43 4.37	1.18 (0.98-1.42) 1.01 (0.83-1.23) Reference	0.41 0.37 1.00	0.42 (0.25-0.70) 0.37 (0.21-0.64) Reference			Eikelboom et al^25^ RE-LY trial Prospective, randomized, double-blind, multicenter trial	December 2005 to December 2007
N = 6,215 Rivaroxaban, n = 3,111 Warfarin, n = 3,104	Rivaroxaban 20 mg^a^ once daily Warfarin	4.86 4.40	1.11 (0.92-1.34) Reference	0.66 0.83	0.80 (0.50-1.28) Reference	0.28 0.61	0.45 (0.23-0.87) Reference	Halperin et al^26^ ROCKET AF trial Prospective, randomized, double-blind, multicenter trial	December 2006 to June 2009
N = 5,678 Apixaban, n = 2,850 Warfarin, n = 2,828	Apixaban 5 mg^b^ twice daily Warfarin	3.33 5.19	0.64 (0.52-0.79) Reference	0.43 1.29	0.34 (0.20-0.57) Reference	0.13^e^ 0.41^e^	0.33^e^ (0.13-0.82) Reference	Halvorsen et al^27^ ARISTOTLE trial Prospective, randomized, double-blind, multicenter trial	December 2006 to April 2010
N = 8,474 Edoxaban 60 mg/30 mg^c^, n = 2,848 Edoxaban 30 mg/15 mg^c^, n = 2,806 Warfarin, n = 2,820	Edoxaban 60 mg/30 mg^c^ once daily Edoxaban 30 mg/15 mg^c^ once daily Warfarin	4.0 2.3 4.8	0.83 (0.70-0.99) 0.47 (0.38-0.58) Reference	0.5 0.4 1.2	0.40 (0.26-0.62) 0.31 (0.19-0.49) Reference	0.3 0.2 0.6	0.46 (0.25-0.84) 0.38 (0.20-0.73) Reference	Kato et al^24^ ENGAGE AF-TIMI 48 trial Prospective, randomized, double-blind, multicenter trial	November 2008 to November 2010

ARISTOTLE = Apixaban for Reduction in Stroke and Other Thromboembolic Events in Atrial Fibrillation (ARISTOTLE); ENGAGE AF-TIMI 48 = Effective Anticoagulation with Factor Xa Next Generation in Atrial Fibrillation–Thrombolysis In Myocardial Infarction 48; RE-LY = Randomized Evaluation of Long-term Anticoagulation Therapy; ROCKET AF = Rivaroxaban Once Daily Oral Direct Factor Xa Inhibition Compared With Vitamin K Antagonism for Prevention of Stroke and Embolism Trial in Atrial Fibrillation.

a 15 mg daily for those with moderately impaired renal function (creatinine clearance 30-49 mL/min)

b 2.5 mg twice daily for patients with 2 or more of the following factors: age ≥80 years, bodyweight ≤60 kg, serum creatinine ≥133 mmol/L (≥1.5 mg/dL)

c For patients with one or more dose reduction criteria: creatinine clearance 30-50 mL/min, body weight 60 kg or less, and/or co-use of potent P-glycoprotein inhibitors.

d The reference was warfarin.

e Includes not only fatal bleeding but also fatal hemorrhagic stroke.

A retrospective meta-analysis and a systematic analysis showed that very elderly patients have improved outcomes with DOACs than with warfarin,^28^ and edoxaban and apixaban are better tolerated than other treatments. However, even with DOAC use, the risk of bleeding appears to increase with age, especially in very elderly patients.

### Frailty and falls

The ENGAGE AF-TIMI 48 (Effective Anticoagulation with Factor Xa Next Generation in Atrial Fibrillation–Thrombolysis In Myocardial Infarction 48) trial found that the risk of stroke and bleeding increased with increased frailty severity.^29^ Similarly, the ARISTOTLE (Apixaban for Reduction in Stroke and Other Thromboembolic Events in Atrial Fibrillation) trial found that patients with a history of falls had a higher risk of major bleeding and death.^30^ No interactions were observed between frailty/fall risks and the safety profiles of DOACs compared with warfarin.

A cohort study in Medicare beneficiaries found that apixaban was associated with lower adverse event rates than warfarin, regardless of frailty status.^31^ In contrast, the FRAIL-AF (Frail Elderly Patients With Atrial Fibrillation) randomized controlled trial found that elderly patients aged ≥75 years (mean, 83 years) with AF and frailty who switched from warfarin to a DOAC (rivaroxaban in approximately half of the patients) had more major or clinically relevant nonmajor bleeding complications than those remaining on warfarin.^32^ It is important to note that there are many validated tools to measure frailty, and, in the absence of a consensus regarding the optimal frailty assessment tool, differences in studies may be expected. Nevertheless, bleeding in patients with AF and frailty remains an important consideration when selecting anticoagulants to prevent stroke.

### Renal impairment

AF and chronic renal impairment frequently coexist, with AF exacerbating renal impairment and renal impairment progression increasing the incidence of AF. Previous studies reported that approximately 30% to 60% of patients with AF had renal impairment.^33^ Patients with AF and renal impairment have higher incidences of stroke and bleeding than patients with normal renal function.^34^ In a subanalysis of the ANAFIE (All Nippon AF In Elderly) Registry (N = 26,202), the incidences of both stroke/systemic embolism and major bleeding increased with progression of renal impairment in Japanese patients with AF aged ≥75 years; DOACs were considered effective and safe even in patients with renal dysfunction (CrCl 15 to <50 mL/min).^35^ A recent meta-analysis of the COMBINE AF (Collaboration Between Multiple Institutions to Better Investigate Non-Vitamin K Antagonist Oral Anticoagulant Use in Atrial Fibrillation) database (including data from the RE-LY (Randomized Evaluation of Long-term Anticoagulation Therapy), ROCKET AF (Rivaroxaban Once Daily Oral Direct Factor Xa Inhibition Compared With Vitamin K Antagonism for Prevention of Stroke and Embolism Trial in Atrial Fibrillation), ARISTOTLE, and ENGAGE AF-TIMI 48 trials) revealed that patients with renal impairment had increased incidences of major bleeding, intracranial hemorrhage, stroke, and death compared with those with normal renal function.^36^ Across continuous CrCl values down to 25 mL/min, the hazard of major bleeding did not change in patients receiving standard-dose DOACs compared with those receiving warfarin. However, all these studies excluded patients with severe renal impairment (CrCl <30 mL/min in the dabigatran, rivaroxaban, and edoxaban studies, and CrCl <25 mL/min in the apixaban study). Therefore, with the exception of 15-mg edoxaban in very elderly patients,^37^ there is a lack of evidence regarding the efficacy and safety of DOACs in patients with severe renal impairment.

### History of bleeding

The ARISTOTLE trial reported that patients with a history of bleeding had a higher risk of major bleeding (adjusted HR: 1.35).^11^ Several prospective observational studies done in elderly and very elderly patients with AF reported a similar finding. A subanalysis of the ANAFIE Registry of AF patients aged ≥80 years, with 89% of patients treated with anticoagulants, revealed that the incidence of major bleeding was higher in patients with a history of major bleeding than in those without (HR: 1.62).^38^ Similarly, the Japanese J-ELD AF Registry (Multicenter Prospective Cohort Study on the Effectiveness and Safety of Apixaban in Japanese Elderly Atrial Fibrillation Patients) revealed that a history of bleeding (HR: 3.81) was associated with bleeding requiring hospitalization in apixaban-treated elderly patients.^39^

### Decline in cognitive function

Cognitive decline and AF are reported to be associated with each other,^40^ and with increased risk for cardiogenic stroke, reduced cerebral perfusion, subcortical white matter lesions, cerebral microbleeds, and asymptomatic stroke.^41^^,^^42^ A subcohort study of the ANAFIE Registry (*N* = 2,963) found that elderly patients with AF and cognitive impairment (*n* = 586) had a significantly higher incidence of stroke and systemic embolism, major bleeding, intracranial hemorrhage, and death (*P* < 0.001),^43^ although no independent effects of cognitive decline on stroke and major bleeding were observed after multivariate adjustment. There remains a lack of evidence from randomized clinical trials regarding how elderly patients with AF and cognitive impairment respond to DOAC treatment.

### Presence of multimorbidities and polypharmacy

Multiple chronic conditions and treatment regimens are common scenarios in elderly and very elderly patients and pose many challenges for health care teams. In such scenarios, there is a high potential for drug–drug interactions and decreased treatment adherence.^44^ Polypharmacy is common in elderly patients with AF, and the use of multiple drugs may adversely affect the efficacy of AF therapies.^45^

In the RE-LY trial, polypharmacy was a risk factor for cardiovascular and bleeding events.^46^ In the ROCKET-AF trial, over half of the patients received more than 5 other medications, and those receiving more medications had a higher risk of bleeding but not stroke.^47^ Given the complexities of managing very elderly patients with AF and multimorbidities/polypharmacy, there is a need for more research regarding the efficacy and safety of treatments in these patients.

### Concomitant antiplatelet therapy

Patients with AF have a high prevalence of chronic coronary artery disease (CAD) as the risk factors for CAD increase with age and often overlap with those for AF.^48^ Whereas oral anticoagulants are needed in patients with AF, antiplatelet agents are often required in patients with CAD.

Analysis of ENGAGE AF-TIMI 48 showed that patients who received concomitant antiplatelet therapy had a similar risk of stroke/systemic embolism, but higher rates of bleeding than those not receiving concomitant antiplatelet therapy.^49^ Analyses of other trials also found an increased risk of major bleeding by concomitant antiplatelet therapy (Table 2).^49^, ^50^, ^51^, ^52^ In ARISTOTLE, a higher percentage of the patients receiving nonsteroidal anti-inflammatory drugs (NSAIDs) at baseline had a history of bleeding than those who had never taken NSAIDs (24.5% vs 15.6%), and incident NSAID use was associated with a higher risk of major bleeding (HR: 1.61).^53^

**Table 2:** Major Bleeding Event Rate in the Presence and Absence of Antiplatelet Drugs

First Author Study	Dose	Antiplatelet + %/Year	Antiplatelet − %/Year	Ratio
Dans et al^50^ RE-LY trial	Dabigatran 150 mg bid	4.41	2.65	1.66
	Dabigatran 110 mg bid	3.94	2.22	1.77
Shah et al^51^ ROCKET AF trial	Rivaroxaban 20 mg daily^a^	4.52	3.11	1.45
Alexander et al^52^ ARISTOTLE trial	Apixaban 5 mg twice daily^b^	3.10	1.82	1.70
Xu et al^49^ ENGAGE AF-TIMI 48 trial	Edoxaban 60 mg/30 mg^c^ once daily	3.55	2.04	1.74
	Edoxaban 30 mg/15 mg^c^ once daily	2.23	1.41	1.58

ARISTOTLE = Apixaban for Reduction in Stroke and Other Thromboembolic Events in Atrial Fibrillation (ARISTOTLE); ENGAGE AF-TIMI 48 = Effective Anticoagulation with Factor Xa Next Generation in Atrial Fibrillation–Thrombolysis In Myocardial Infarction 48; RE-LY = Randomized Evaluation of Long-term Anticoagulation Therapy; ROCKET AF = Rivaroxaban Once Daily Oral Direct Factor Xa Inhibition Compared With Vitamin K Antagonism for Prevention of Stroke and Embolism Trial in Atrial Fibrillation.

a 15 mg daily if creatinine clearance was 30 to 49 mL/min.

b A reduced dose of apixaban 2.5 mg or placebo twice daily was administered to 831 patients (4.7%) with 2 or more of the following criteria: age ≥80 years, weight ≤60 kg, or serum creatinine concentration ≥1.5 mg/dL (133 mmol/L).

c For patients with one or more dose reduction criteria: creatinine clearance 30-50 mL/min, body weight 60 kg or less, and/or co-use of potent P-glycoprotein inhibitors.

In the recent EPIC-CAD (Edoxaban versus Edoxaban with Antiplatelet Agent in Patients with Atrial Fibrillation and Chronic Stable Coronary Artery Disease) trial, patients with AF and CAD (mean age 72 years) treated with edoxaban alone had a lower incidence of all-cause death than those who received edoxaban and an antiplatelet drug.^54^ Moreover, the incidence of major or nonmajor bleeding was lower in patients who received edoxaban alone. Similar findings were reported for the Japanese-specific dose of rivaroxaban.^55^ Thus, it is important to closely monitor patients with AF and comorbid CAD and to avoid the unnecessary use of antiplatelet therapies, particularly in elderly and very elderly patients with AF at risk of bleeding.

## DOAC therapy for very elderly patients with AF

Complicating factors associated with an increased risk of bleeding in very elderly patients, as discussed above, necessitate the careful selection of DOACs (Table 1).^24^, ^25^, ^26^, ^27^ A post hoc analysis of the landmark clinical trials revealed a significant treatment effect modification by age in the safety of dabigatran and rivaroxaban, but no interaction with age in the safety of apixaban and edoxaban, even in patients aged ≥80 years, compared with warfarin.^56^ Although these findings are not derived from head-to-head comparisons of DOACs, this may inform the selection of DOACs for very elderly patients at high risk of bleeding.

A recent post hoc analysis of ENGAGE AF-TIMI 48 evaluated the effects of edoxaban in very elderly patients aged ≥80 years (mean age 83 years) without dose-reduction criteria, receiving 60 mg edoxaban (higher-dose group) vs 30 mg edoxaban (lower-dose group).^57^ Major bleeding events, especially major gastrointestinal bleeding events, were lower in patients receiving the lower dose than in those receiving the higher dose, with no increase in ischemic stroke. Very elderly patients receiving the lower dose had a median trough concentration similar to that in patients aged <80 years receiving the higher dose and achieved a similar degree of inhibition of endogenous factor Xa at trough. These findings regarding the effects of lower-dose DOACs in very elderly patients are important; however, it is important to note that the off-label use of lower-dose DOACs should be justified.

Importantly, patients at high bleeding risk, such as those with CrCl <25 to 30 mL/min, were excluded from the landmark trials, and therefore there has been a lack of evidence for most DOACs in this patient group. The ELDERCARE-AF (Edoxaban Low-Dose for Elder Care Atrial Fibrillation Patients) trial evaluated the efficacy and safety of very low-dose edoxaban (15 mg once daily) in very elderly patients with AF and high bleeding risk.^37^ Included patients were those ineligible for standard oral anticoagulant therapy at approved doses, and who had at least one bleeding risk factor (CrCl 15-30 mL/min, bodyweight ≤45 kg, a history of bleeding from the critical organs, and concomitant use of NSAIDs or antiplatelet therapy). Very low-dose edoxaban-treated patients had a reduced risk of stroke/systemic embolism compared with placebo (HR: 0.34), and a nonsignificant increase in major bleeding (HR: 1.87; *P* = 0.09).^37^ There was no fatal bleeding and no increase of intracranial hemorrhage with edoxaban, but a substantial increase in gastrointestinal bleeding with edoxaban (HR: 2.85). Thus, very low-dose edoxaban may be an option in very elderly patients with high bleeding risk who are ineligible for standard oral anticoagulation therapy. When stratified by age,^58^ frailty/nonfrailty,^59^ bodyweight,^60^ and renal function subgroups,^61^ the beneficial effect of edoxaban had no interaction with any of these complicating factors. However, the risk of major bleeding events increased in patients aged ≥90 years and those with CrCl 15 to 29 mL/min, further highlighting the caution required in these patient groups. A pharmacokinetic subanalysis showed that the plasma concentration of very low-dose edoxaban (15 mg) at trough in patients with CrCl 15 to 29 mL/min was 1.65 times higher than in patients with CrCl ≥30 mL/min.^62^ This may explain the therapeutic effect of very low-dose edoxaban and increased bleeding in the very elderly patients with severe renal impairment. Further studies from a standpoint of precision medicine, including the use of other DOACs and for patients of different ethnicities, are necessary.

## Modifiable measures for preventing adverse events in very elderly patients

Because stroke risk increases with age, stroke prevention with oral anticoagulants, including DOACs, remains one of the most important pharmacotherapy strategies. Considering the complicating factors that lead to difficulties in the use of oral anticoagulants—especially in very elderly patients—physicians have an important role in lowering the risk of major adverse effects of oral anticoagulants (including major bleeding, particularly intracranial hemorrhage and fatal bleeding). Measures that can be taken include modification and/or treatment of risk factors that may increase the risk of major bleeding (Central Illustration). First, blood pressure should be controlled in accordance with clinical guidelines. By assessing blood pressure successively measured at home for 1 week, the ANAFIE Registry subcohort study^63^ (mean age 81.4 years) demonstrated that the incidences of stroke, major bleeding, and intracranial hemorrhage all significantly increased with increasing home systolic blood pressure. Compared with patients with an average home systolic blood pressure <125 mm Hg, blood pressure ≥145 mm Hg was associated with increased risks of these events. Second, the type of DOAC should be carefully selected and managed, considering the metabolism of each DOAC and potential drug–drug interactions. Third, all coadministered medications should be reassessed both to evaluate their necessity and to check for possible interactions with oral anticoagulants; the number of medications should be reduced as much as possible to prevent polypharmacy. Fourth, the use of antiplatelet drugs should be reconsidered in patients treated with oral anticoagulants, and—if possible—the antiplatelet drug should be discontinued. Fifth, for patients with a history of bleeding, the cause of bleeding should be treated to reduce the risk of rebleeding during oral anticoagulant therapy. Sixth, periodic observation of renal and liver function should be performed; if renal or hepatic impairment is identified, its cause should be found and treated. If the cause cannot be found or treated, the type and dose of oral anticoagulant should be reviewed, and the use of a low-dose anticoagulant, such as very low-dose edoxaban, may be considered if approved DOAC doses cannot be tolerated. Seventh, although frailty remains a difficult clinical challenge, any contributing factors, such as low physical activity and low bodyweight, need to be addressed if possible, and the risk of falls should be minimized. When major bleeding occurs or nonmajor bleeding reoccurs even with application of these approaches, use of the currently available oral anticoagulant should be withdrawn and other measures, such as a left-atrial appendage closure or resection of the left-atrial appendage, may be considered.

## Summary and conclusions

This review of the evidence regarding the use of DOACs to improve health outcomes in elderly and very elderly patients with AF shows that such patients are an under-researched group, particularly those with a history of bleeding, severe renal impairment, cognitive decline, and frailty. These patients are often excluded from randomized controlled trials, and an important limitation of this review is the relative lack of randomized controlled trials in these patient groups. Although a knowledge gap remains, new evidence has begun to fill this gap.

## Funding support and author disclosures

Medical writing was supported by 10.13039/501100002973Daiichi Sankyo Co, Ltd, Japan. Dr Okumura has received lecture fees from Daiichi Sankyo Co, Ltd, Nippon Boehringer Ingelheim Co, Ltd, Bristol Myers Squibb K.K., Medtronic Japan Co, Ltd, and Johnson & Johnson K.K., outside the submitted work. Dr Akao has received research funding from 10.13039/100015731Bayer Yakuhin Ltd and 10.13039/501100002973Daiichi Sankyo Co, Ltd, and remuneration from Bristol Myers Squibb K.K., Nippon Boehringer Ingelheim Co, Ltd, Bayer Yakuhin Ltd, and Daiichi Sankyo Co, Ltd, outside the submitted work. Dr Suzuki has received research funding from 10.13039/501100002973Daiichi Sankyo Co, Ltd, and remuneration from Bristol Myers Squibb K.K. and Daiichi Sankyo Co, Ltd, outside the submitted work. Dr Yamashita has received research funding from 10.13039/501100002973Daiichi Sankyo Co, Ltd, manuscript fees from Daiichi Sankyo Co, Ltd and Bristol Myers Squibb K.K., and remuneration from Daiichi Sankyo Co, Ltd, Bayer Yakuhin Ltd, Bristol Myers Squibb K.K., Nippon Boehringer Ingelheim Co, Ltd, Novartis, and Ohtsuka Pharmaceutical, Co, Ltd, outside the submitted work.
